# Secretoneurin plasma levels are decreased after catheter ablation for atrial fibrillation—patients with AF produce lower SN levels than healthy individuals: the SAFE registry

**DOI:** 10.3389/fcvm.2025.1664855

**Published:** 2025-10-16

**Authors:** Jiří Plášek, Jiří Vrtal, Nela Chobolová, Zdeněk Švagera, Diana Drieniková, Jiří Pudich, Martin Ráchela, David Stejskal, Jozef Dodulík, Jan Václavík

**Affiliations:** ^1^ Department of Internal Medicine and Cardiology, University Hospital Ostrava, Ostrava, Czechia; ^2^ Research Center for Internal and Cardiovascular Diseases, Faculty of Medicine, University of Ostrava, Syllabova, Czechia; ^3^ Institute of Laboratory Medicine, University Hospital Ostrava, Ostrava, Czechia; ^4^ Institute of Laboratory Medicine, University of Ostrava, Ostrava, Czechia

**Keywords:** secretoneurin, caMKII, calmodulin, atrial fibrillation, catheter ablation

## Abstract

**Background:**

Secretoneurin (SN) is a neuropeptide that counterbalances disorders of cell calcium cycling via calmodulin and thus may be involved in arrhythmogenesis. We aimed to associate plasma standard biomarkers and SN levels before, during, and after catheter ablation for atrial fibrillation (AF) with various clinical and paraclinical variables, foremost AF recurrence.

**Methods:**

We prospectively enrolled consecutive patients undergoing catheter ablation for AF in University Hospital Ostrava between March 2023 and January 2024. SN was analyzed from venous, left atrial, and coronary sinus blood with the ELISA method. Plasma SN levels were compared before, during, and after ablation and with those of healthy individuals, with nonparametric tests. ClinicalTrials.gov ID: NCT05794464.

**Results:**

A total of 137 patients (64% males, age 63 ± 11) and 34 healthy individuals (38% males, mean age 31 ± 7.1) were included in the analysis. Plasma SN levels were significantly lower after the catheter ablation as compared with SN levels before (34.0 ± 15.1 and 40.0 ± 17.3 pmol/L, *p* = 0.032). Healthy individuals produced higher plasma SN levels (50.7 ± 15.3 pmol/L) as compared with those in patients with AF both before and after catheter ablation (*p* = 0.0068, *p* < 0.001). Furthermore, lower postprocedural levels of SN were associated with AF recurrence (*p* = 0.035). The C-statistic for SN after the procedure to separate presence or absence of AF recurrence was 0.65 with bootstrap 95% CI 0.5–0.8. Of note, SN after the procedure correlated with plasma NT-proBNP levels (*p* < 0.001). PFA caused greater myocardial damage than did RFA (*p* < 0.001); inflammatory markers were increased post procedurally in both the RFA and the PFA group. Subclinical intravascular hemolysis occurred in the PFA group.

**Conclusion:**

Plasma secretoneurin levels are decreased after catheter ablation for atrial fibrillation; patients with AF produce lower SN levels than healthy individuals. Lower postprocedural levels of SN are associated with AF recurrences.

## Introduction

Atrial fibrillation (AF) is the most common arrhythmia, and substantial resources are being spent on understanding the detailed mechanism underlying AF, its causes, diagnostics, and treatment (1). After decades of research, the causes and course of the disease are still not completely understood. Therefore, different predictors of both new-onset and recurrent arrhythmia are needed. Ca^2+^ is a fundamental second messenger in all cell types, including cardiomyocytes, and is required for numerous essential cellular functions (2). Dysregulation of cytosolic and mitochondrial Ca^2+^ occurs in heart failure and AF (2). Abnormal Ca^2+^ handling is critical in the induction and maintenance of AF, contributing to both ectopic activity and AF-maintaining re-entry circuits (3). The most common cause of abnormal calcium handling in the cardiomyocyte is dysfunction of ryanodine receptor 2 (RYR2), which in its hyperphosphorylated state leads to Ca^2+^ leakage from the sarcoplasmic reticulum (SR) (2). Secretoneurin (SN) is indirectly involved in calcium handling through calmodulin and may be a novel cardiovascular, especially arrhythmia-associated, biomarker (4). Secretoneurin is a 33-amino-acid neuropeptide from the chromogranin peptide family. Its cell signaling pathway differs from those of both N-terminal pro natriuretic peptide (NT-proBNP) and troponin (5). SN's main intracellular action is inhibition of calmodulin-dependent kinase II (CaMKII), which may be cardioprotective. Myocardial CaMKII inhibition leads to improved excitation–contraction coupling and arrhythmia susceptibility attenuation by suppressing Ca^2+^ leakage from the sarcoplasmic reticulum (6). SN is very probably upregulated in pro-arrhythmogenic states, counterbalancing increased calcium leakage from the sarcoplasmic reticulum mostly due to RYR2 hyperphosphorylation (5, 6). Myocardial pro-SN expression was increased in CPVT mice. Furthermore, SN levels were elevated in patients with CPVT and following ventricular arrhythmia–induced cardiac arrest (6).

SN has not been studied in the AF patient population, nor in any relation to catheter ablation (CA). Therefore, we aimed to investigate the dynamics of SN plasma levels before and after catheter ablation also with respect to the technology used and presence/absence of AF episodes, the differences between coronary sinus and left atrial SN plasma levels, and also comparison to the SN plasma levels in healthy individuals. Moreover, we aimed to study the differences between paroxysmal and persistent AF including associations with AF episodes and with other standard cardiovascular biomarkers, such as NT-proBNP, troponin, markers of inflammation and markers of intravascular hemolysis in the pulsed-field ablation group since this is of importance regarding the safety issues.

## Materials and methods

### Patients

For this study, we prospectively enrolled consecutive patients undergoing catheter ablation for AF in University Hospital Ostrava between March 2023 and January 2024. The inclusion and exclusion criteria were prespecified, and the study was registered before its start at ClinicalTrials.gov (identifier: NCT05794464), as Secretoneurin as a biomarker of Atrial Fibrillation rEcurrence after catheter ablation, the prospective SAFE registry.

The study sample comprised patients with paroxysmal and persistent AF: permanent and/or long-standing (>24 months) persistent AF was an exclusion criterion. Patients had to be indicated for catheter ablation at the time of screening, before blood sampling was performed and informed consent was signed. Patients with known heart failure with reduced ejection fraction were excluded, except those with tachycardia-induced heart failure in causal relation to AF. Cerebral ischemia and/or stroke less than 3 months prior, severe kidney injury or severe renal insufficiency (end-stage kidney disease 5), hepatic insufficiency (limited biomarker sampling, i.e., >5-fold elevated aminotransferase) and myocardial infarction <3 months prior were all exclusion criteria. In general, except for reduced ejection fraction, there were no other exclusion criteria only those interfering either with laboratory diagnostics or ablation procedure itself, patients were not preselected but consecutively enrolled. This study was approved by the Institutional Review Board of University Hospital Ostrava (Nr. 128/2023) and conducted in accordance with the Helsinki Declaration. All patients gave signed informed consent.

### Ablation procedure

Bilateral femoral veins were punctured under direct ultrasound (US) guidance in all patients. In the most common setting, two Swartz^™^ SL1 8.5 Fr sheaths were introduced via the right femoral vein and two short sheaths via the left femoral vein: 7 Fr for coronary sinus catheter and 11 Fr for intracardiac echocardiography probe. Alternatively, Agilis^™^ medium sweep sheath was used instead of the second Swartz^™^ SL1 sheath. In the pulsed-field ablation (PFA) setting, only one Swartz^™^ sheath deployed in the right groin was used for transseptal puncture and then switched for 16.8F outer diameter Faradrive^™^ sheath.

No arterial access was used for procedure monitoring. The sheaths were removed at the end of the procedure and venous hemostasis was achieved by “Z”-stitch followed by 6-hour 1,000 g sandbag compressions. Bed rest was implemented in all patients till the next morning.

Intracardiac echocardiography was used throughout the whole procedure as an institutional standard. It was used particularly for guiding the transseptal puncture, determining accurate antral ablation points, tagging the course of the esophagus, titration of radiofrequency energy, and early detection of complications.

CA was performed with a 3.5 mm irrigated-tip catheter (Navistar® Thermocool, Thermocool Smarttouch® Biosense Webster, Diamond Bar, CA, USA or TactiCath^™^, Abbott, St. Paul, MN, USA). The catheter was navigated with the use of 3D electroanatomic system (CARTO®; Biosense Webster or Ensite Precision®; Abbott, St Paul, MN, USA). Linear point-by-point lesions were placed around the ostia of pulmonary veins with the endpoint of electrical isolation. In patients with the advanced atrial disease, empirical linear lesions and/or superior vena cava isolation and/or biatrial electrogram-guided ablation was performed. Cavotricuspid isthmus was ablated in 20.5% of procedures. Mappable atrial tachycarrhythmias (ATs) were always targeted. The noninducibility of arrhythmia was the desired end point of repeated procedures.

Radiofrequency energy was delivered by an EP Shuttle^™^ (Stockert, Freiburg, Germany) or Smartablate^™^ (Biosense Webster) or Ampere^™^ generator (St Jude Medical). Constant irrigation flow of 20 ml/min (30 ml/min inside the coronary sinus) through a Cool Flow® pump (Biosense Webster) or Cool Point^™^ (St Jude Medical), was employed. The power mode was used with a preset power up to 25–35 W and downregulation when the tip temperature of 43°C was achieved. Power output was mostly reduced to 20–25 W at the posterior wall of the left atrium (LA) and inside the coronary sinus. The energy was delivered either in a point-by-point fashion (20–30 s at one spot) or by dragging.

Pulsed-field ablation was in contrast to radiofrequency ablation performed under general anesthesia and without an electroanatomical mapping system; only a Farapulse PFA system (Boston Scientific, Marlborough, MA, USA) was used. A Farawave^™^ catheter (31 mm, in one case 35 mm) was, via a Faradrive^™^ sheath, introduced into the left atrium and navigated over-the-wire to the ablation site. Pulsed-field energy was delivered by a Farastar^™^ generator with a voltage output of 1.8–2 kV. Energy applications were delivered as a biphasic waveform on a microsecond scale, unsynchronized to cardiac rhythm. A group of five consecutive pulse trains accounting for 2.5 s of ablation time constituted one PFA application. The PFA PVI protocol per one pulmonary vein was as follows: 2 applications in the “basket shape”, rotation of the basket and another 2 applications following 2 + 2 applications in the “flower” conformation with rotation in between these applications. Finally two “anchor” lesions per vein were added to the posterior wall. At the end of the application set all veins were checked for reconnection. If reconnection occurred, at least two applications per site were added.

### Perioperative anticoagulation

All procedures were performed on uninterrupted warfarin with the target INR between 2 and 3 or minimally (single dose) interrupted direct oral anticoagulants. After the procedure, either direct oral anticoagulants or warfarin was restarted after a drop in the ACT to under 170 s.

Anticoagulation treatment continued for at least 3 months after the procedure in low-risk patients or lifelong in high-risk patients according to the ESC guidelines (1).

### Holter ECG, follow-up

Patients were followed for AF recurrence: all patients had a 24-hour ECG Holter and external loop recorder (ELR Medical Data Transfer, Brno, Czech Republic) for 2 weeks after the 3 months of the blanking period. If no AF was detected, another ELR recording followed after 3 months to enable possible anticoagulation discontinuation in patients with low thromboembolic risk. Clinical outpatient control ensued in the 4th and 8th months. AF recurrence was defined as any atrial fibrillation, atrial flutter or atrial tachycardia episode longer or equal to 30 s detected on either ECG Holter, ELR or presence of AF on 12-lead ECG.

### Blood sampling

All blood samples were obtained from patients in the fasting state. The first blood samples were obtained before the procedure, from the antecubital vein. The second blood sample was obtained the next morning after the procedure, also from the antecubital vein. In a subset of 67 patients, blood was also drawn by SL1 sheath directly from the left atrium and then from the coronary sinus. The SL1 sheath was “on wire” navigated to the ostium of the coronary sinus, where the decapolar catheter was placed. In all samples, the first approximately 3–5 ml were discarded.

### Secretoneurin analysis

SN was analyzed from venous (arterial in case of the LA site) blood by use of the ELISA method (CardiNor AS, Oslo, Norway) (7). The blood was drawn into lithium–heparin tubes, and the plasma was separated and frozen to −70°C; only one defrosting cycle was allowed. The intra-assay coefficients of variation for SN were lower than 5% and the interassay coefficient was lower than 10%. The level of determination (LoD) for the CardiNor SN is 5.1 pmol/L, the level of quantification (LoQ) is 7.6 pmol/L, and the analytical range is 11.8–299.2 pmol/L.

### Biochemical analysis

Bilirubin, haptoglobin, hemopexin, and lactate dehydrogenase (LDH) were tested at the Institute for Laboratory Medicine, University Hospital Ostrava. Plasma LDH and bilirubin levels was tested using commercial absorption spectrophotometry (Atellica Solution, Siemens, USA). Haptoglobin and hemopexin were measured using a commercial immunonephelometric assay (Atellica Neph 630, Siemens, USA). NT-proBNP and troponin I plasma levels were tested using a commercial immunomethod with chemiluminescent detection (Atellica Solution, Siemens, USA). Complete blood counts with differential counts and reticulocytes were measured automatically by use of a Sysmex XN (XN-9000, Sysmex Corporation, Japan).

### Statistical analysis

Continuous variables are expressed as means ± standard deviation and compared by *T*-test or Mann–Whitney *U*-test, as appropriate. Categorical variables are expressed as percentages and compared by the chi-square test, Fisher exact test, or logistic regression if appropriate. The association of SN pre- and postprocedural values was investigated by linear regression analysis. The differences between pre- and postprocedural SN and other continuous variables were assessed by the non-parametric Wilcoxon paired test. The differences between SN preprocedural, SN postprocedural, and healthy individuals’ values was compared by non-parametric Kruskal–Wallis test. Differences in SN values categorized by the presence/absence of different factors were assessed by Kruskal–Wallis test. Also strict uni- and multivariate logistic regression was used to assess association of all biomarkers including SN in relation to postablation AF episodes. For time-to-event analysis of AF recurrence in two major subgroups of AF, Kaplan–Meier curves were plotted. The C statistic was calculated for the postprocedural SN to separate the presence and absence of postablation AF episodes. Two-tailed α < 0.05 was considered statistically significant, except for the test of equality of covariance matrices where p < 0.005 was considered significant. All analyses were performed using IBM SPSS for MAC version 23 (IBM, New York, USA), or RStudio Posit Cloud version 4.3.2 (Posit Software, PBC, Boston, USA). The data visualization was done exclusively in RStudio 4.3.2 (Posit Software, PBC, Boston, USA).

## Results

A total of 137 patients (64% males, age 63 ± 11 years) and 34 healthy individuals (38% males, mean age 31 ± 7.1; healthy cohort published previously by our group) (8) were included in the analysis. For baseline characteristics, see Table 1. Procedural success in terms of PV isolation was 100% in both the PFA and the RFA group. Current mean follow-up was 10 ± 3 months. There were no meaningful differences between males and females, except for anthropometric variables.

**Table 1:** Baseline characteristics of the study population, males vs. females.

Factor	Total population	Males	Females	*P* value
	*N* = 137	*N* = 88	*N* = 49	
Age (years)	63 ± 11	61.2 ± 11.2	66.1 ± 10	*P* = 0.01
Males (%)	64	–	–	
Body weight (kg)	79.6 ± 37.5	88.1 ± 38	64.7 ± 32.1	*P* < 0.001
Body height (cm)	174.4 ± 10.6	180.4 ± 7.5	164 ± 6.3	*P* < 0.001
Body mass index (kg/m^2^)	30 ± 5.1	30.2 ± 5.2	30 ± 5.1	*P* = 0.94
LV ejection fraction (%)	56.6 ± 9.2	55.6 ± 9.6	58.5 ± 8.3	*P* = 0.1
LA volume index (ml/m2)	36.8 ± 19	36.7 ± 16	37 ± 24	*P* = 0.94
Left atrial diameter (mm)	43.8 ± 6	44.8 ± 5.3	42 ± 6.7	*P* = 0.02
Hypertension (%)	79.7	81	77.6	*P* = 0.92
Parox. atrial fibrillation (%)	69.6	67.4	73.5	*P* = 0.47
Persist. atrial fibrillation (%)	30.4	32.6	26.5	*P* = 0.46
Dyslipidemia (%)	67.7	72.6	59.2	*P* = 0.11
Diabetes mellitus (%)	13	17	26.5	*P* = 0.07
Previous stroke/TIA (%)	12	13.1	10.2	*P* = 0.62
Coronary artery disease (%)	22.6	23.3	20.4	*P* = 0.65
ACEi (%)	34.6	31.8	38.3	*P* = 0.45
Betablockers (%)	82.7	80	87.2	*P* = 0.29
AT1 blockers (%)	26.3	27.1	25.5	*P* = 0.65
Propafenon (%)	36.8	35.3	40.4	*P* = 0.56
Amiodaron (%)	30.8	31.4	27.7	*P* = 0.63
Sotalol (%)	6.8	9.3	2.1	*P* = 0.23
Diuretics (%)	6.3	5.9	8.5	*P* = 0.57
DOACs (%)	94.2	95.3	91.5	*P* = 0.78
Warfarin (%)	3.8	2.4	6.4	*P* = 0.62
Reduced DOACs dose (%)	4	3.6	4.3	*P* = 0.84
Statin (%)	56.8	62.4	47.8	*P* = 0.11

Indices are shown as mean ± standard deviation or proportion in percentages and compared for males and females. ACEi, angiotensin-converting enzyme inhibitor;AT1, Angiotensinogen II receptor type 1; DOAC, direct oral anticoagulant; LV, left ventricle; NYHA, New York Heart Association; TIA, transient ischemic attack.

**Figure 1:**
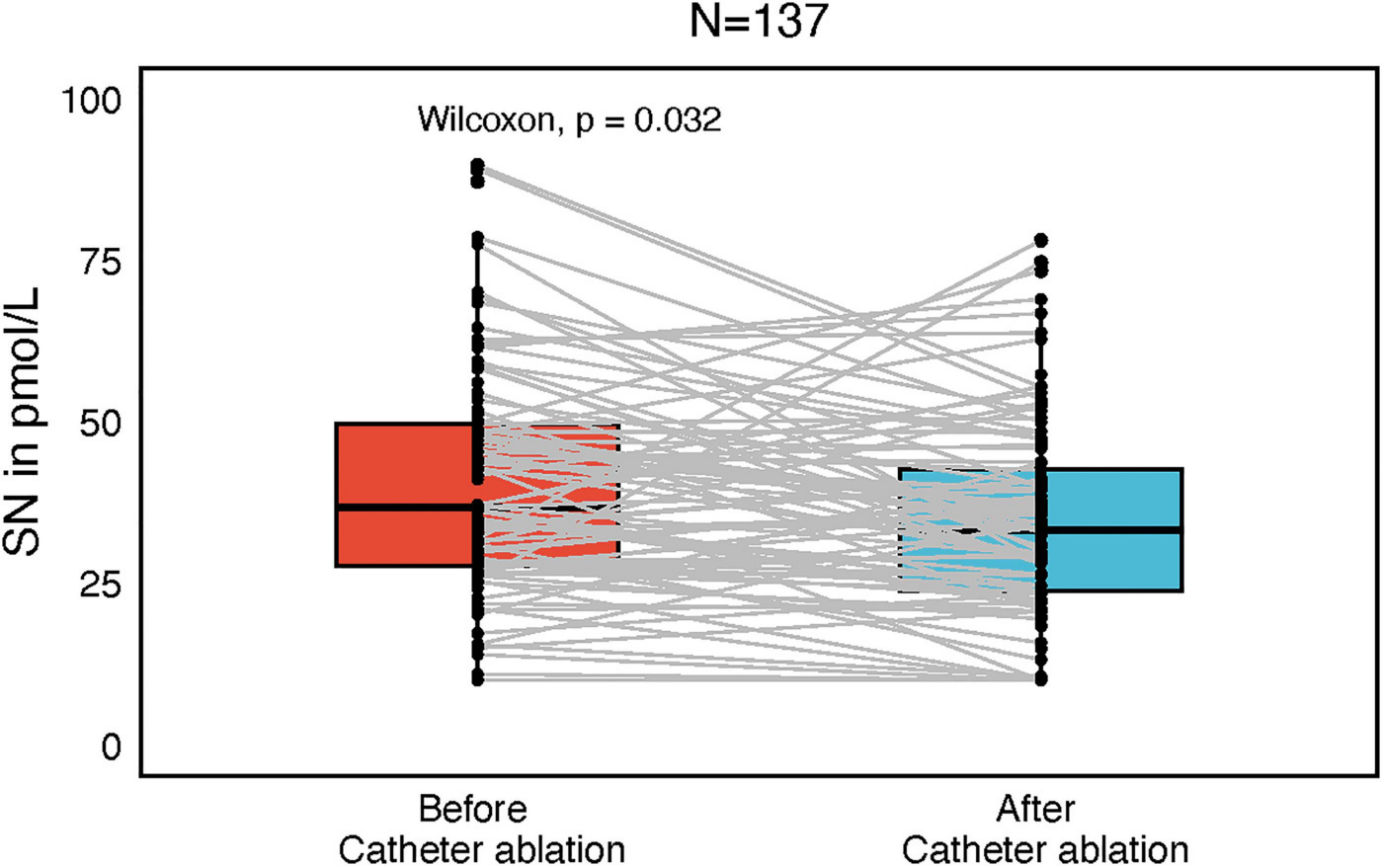
Box plot with central jitter reflecting particular secretoneurin (SN) values in pmol/L, compared before and after catheter ablation.

**Figure 2:**
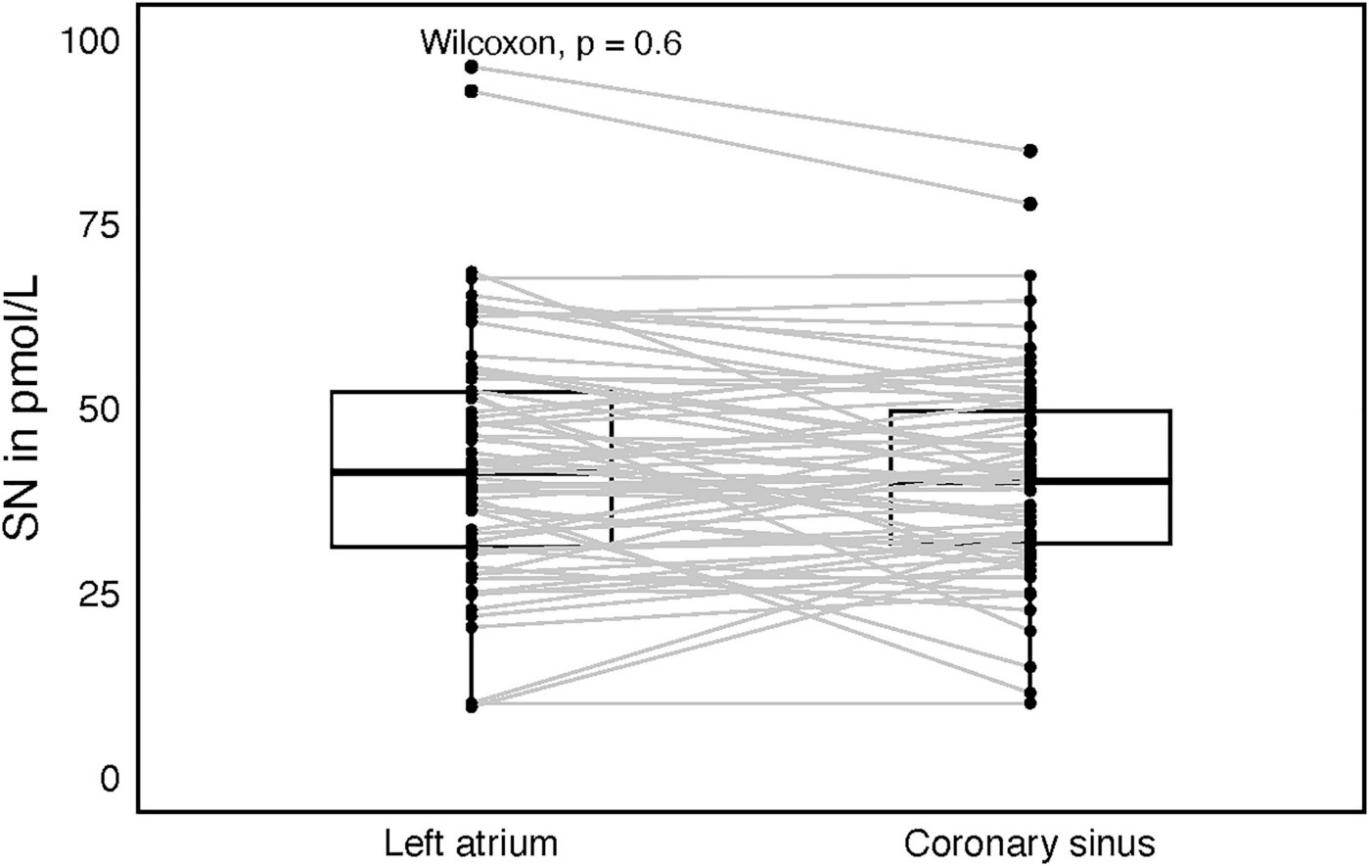
Box plot with central jitter comparing secretoneurin (SN) values in the left atrium and the coronary sinus.

Plasma SN levels were significantly lower after the catheter ablation (CA) as compared to SN levels before CA (34 ± 15.1 and 40 ± 17.3 pmol/L, *P* = 0.032, Figure 1). There was no difference in SN plasma levels before and after CA when categorized by arrhythmia type (paroxysmal/persistent), initial rhythm (sinus, *N* = 90 vs. AF, *N* = 47) or sex, although in females a trend towards higher postprocedural SN levels was observed. Also, separate subgroup analyses of males/females and arrhythmia types revealed no difference in SN plasma levels before and after the procedure. In a subgroup of 67 patients (70.2% males), who were sampled also for LA and coronary sinus (CS) plasma SN levels, no difference was found between these two sites (42.8 ± 16.7, 40.9 ± 14.1 pmol/L, Figure 2).

**Figure 3:**
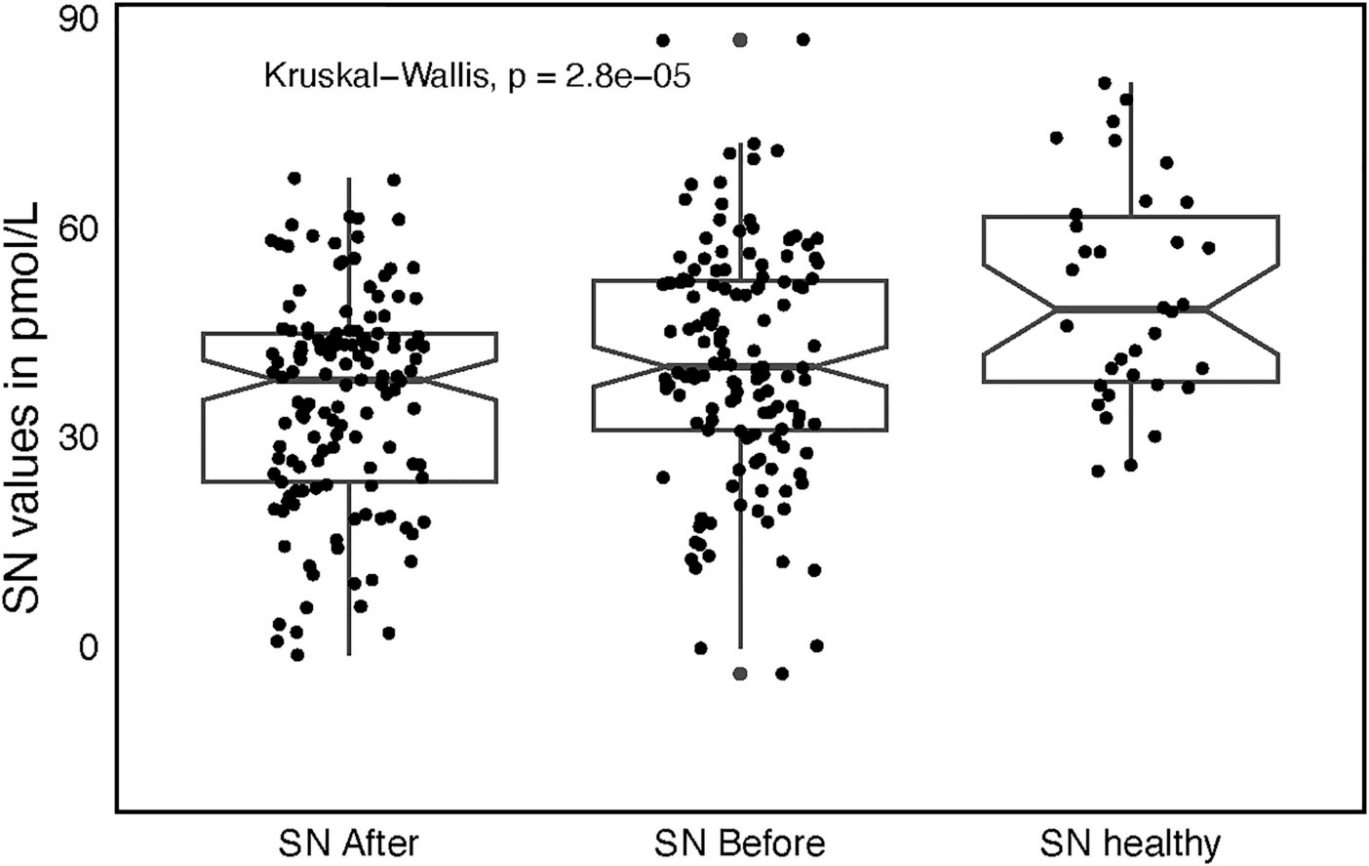
Box plot with jitter comparing secretoneurin (SN) values among three groups, before catheter ablation (SN before), after catheter ablation (SN after), and in healthy individuals (SN healthy) with accompanying significance across the groups.

Healthy individuals produced higher SN plasma levels (50.7 ± 15.3 pmol/L) as compared to SN levels both before and after catheter ablation sampled from patients with AF (*P* = 0.0068, *P* < 0.001, respectively, Figure 3). This holds also true for the SN plasma levels stratified by quartiles of age, only Q1 (*N* = 34) was analyzed to diminish the age gap between healthy individuals and AF study group. Q1 quartile SN1 levels were of 39.2 ±  14.1 and SN2 levels of 32.2 ± 12.7 pmol/, respectively. Both were significantly lower as compared to the healthy individuals (*P* = 0.002, *P* < 0.0001).

These findings persisted after stratifying SN by age quartiles. To minimize the age difference between healthy controls and the AF cohort, we restricted the comparison to the youngest quartile (Q1; *n* = 34). Mean SN1 and SN2 levels in Q1 were 39.2 ± 14.1 and 32.2 ± 12.7 pmol/L, respectively—both significantly lower than in healthy controls (SN1: *P* = 0.002; SN2: *P* < 0.0001).

In a *post hoc* power analysis (α = 0.05), statistical power was 100% for comparing post-ablation SN levels with healthy controls, 94.4% for pre-ablation vs. healthy controls, and 86.4% for pre- vs. post-ablation.

**Figure 4:**
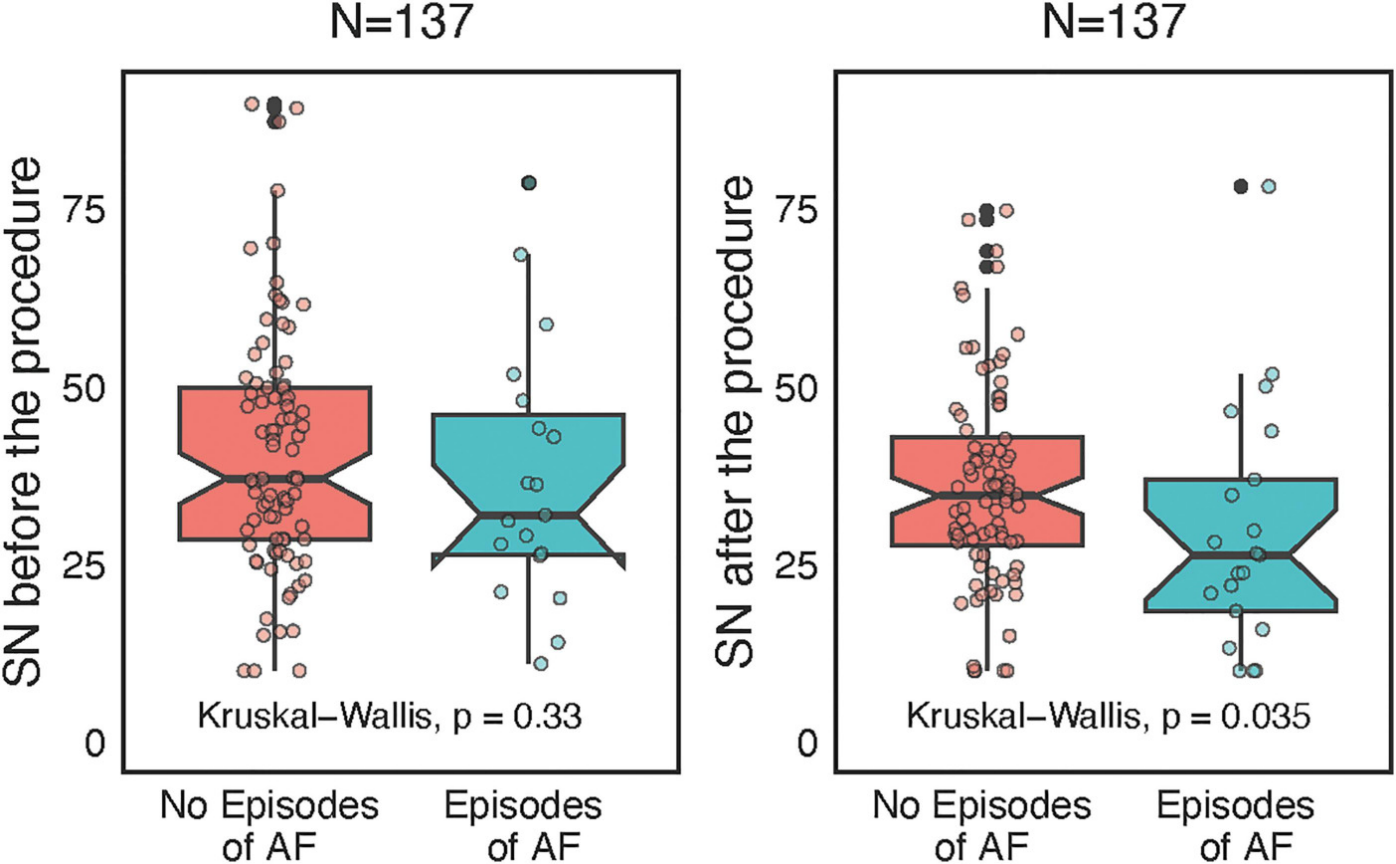
Wilcoxon paired-test separate analyses before and after catheter ablation according to atrial fibrillation (AF) recurrences, represented as box plots with jitter and interconnected grey lines.

Furthermore, lower postprocedural levels of SN were associated with AF recurrence (*p* = 0.035, Figure 4). A 5 pmol/L increment of postprocedural SN decreased 0.9× the risk of AF recurrence (OR 0.93, 95% CI 0.74–1.3, *P* = 0.049). There were no meaningful differences between the AF recurrence and nonrecurrence groups except for body weight and height (Supplementary Table 1). The C-statistic for SN after the procedure to separate the presence or absence of AF recurrence was 0.65 with bootstrap 95% CI 0.5–0.8. The cutoff value assessed by the Youden index was 34.7 pmol/L, the sensitivity 54%, and specificity 68%. A postprocedural value higher than 34.7 pmol/L increased the chance of staying in sinus rhythm, but both the sensitivity and specificity were very low. Therefore, postprocedural SN had limited predictive capacity regarding the AF recurrence, which was confirmed by the strict univariate logistic regression model, where was only trend towards it's predictive capacity in relation to AF episodes (see Supplementary Material). Postablation AF episode rate was 25/137 (18.2%) in the whole group, but 18/95 (18.9%) in the paroxysmal AF subgroup and 7/42 (16.7%) in the persistent AF subgroup. After the blanking period of 90 days the AF recurrence rate was 9/95 (9.5%) in the paroxysmal AF subgroup, no recurrencies were observed in the persistent AF subgroup.

**Figure 5:**
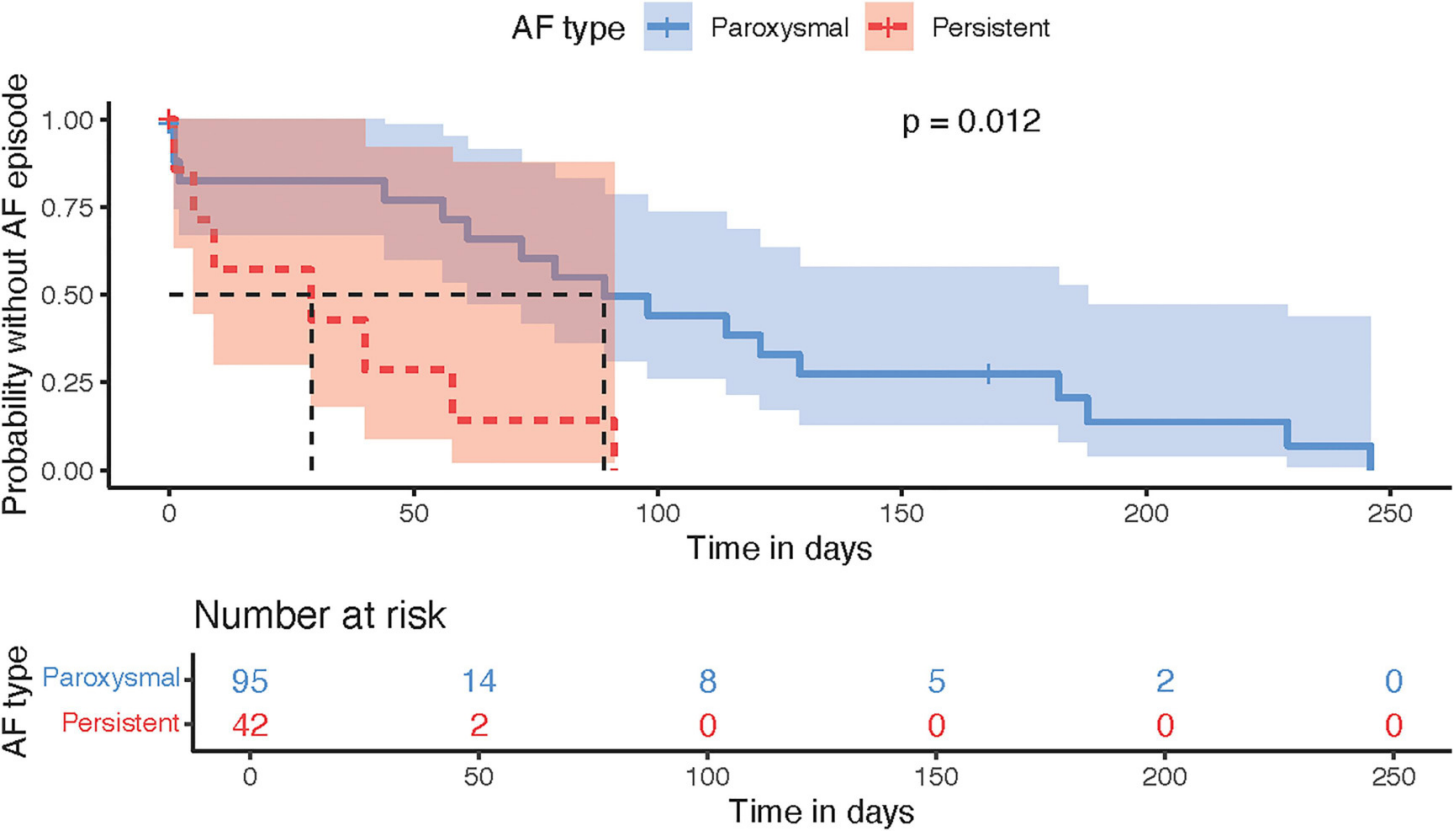
Kaplan–meier time-to-event analysis plot displaying the comparison of paroxysmal vs. persistent AF according to the probability of AF episode.

**Figure 6:**
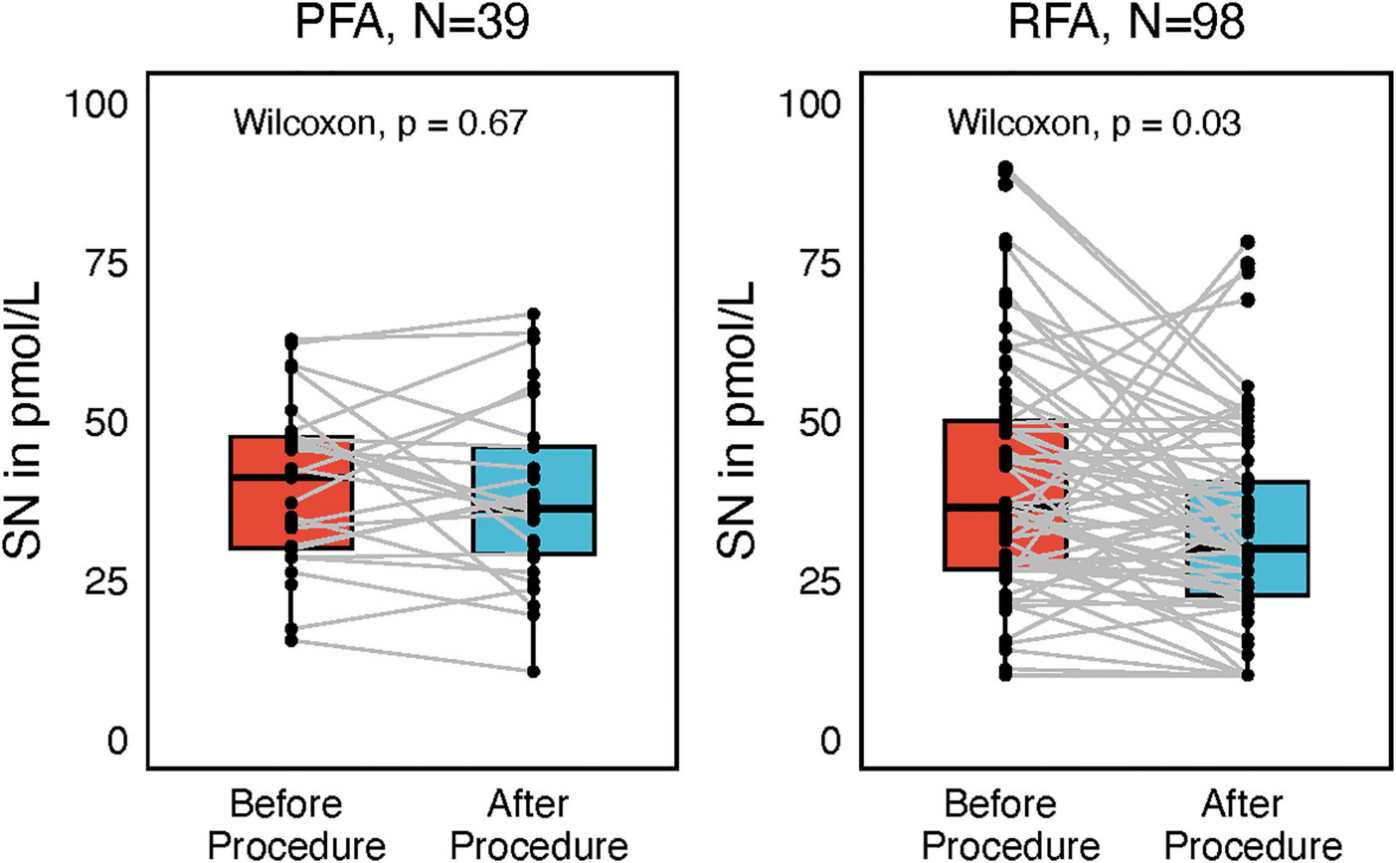
Separate subgroup analysis by ablation modality, either pulsed-field ablation (PFA) or radiofrequency ablation (RFA), comparing secretoneurin (SN) values before and after catheter ablation, represented as box plots with jitter.

AF episodes were somewhat higher in the paroxysmal AF, but the time to episode was longer than in persistent AF (*P* = 0.012, Figure 5). Subgroup analysis by ablation modality showed a difference in plasma SN levels before and after ablation only in the radiofrequency subgroup (*P* = 0.03, Figure 6), although this could have been caused by the significantly smaller sample size of the PFA group (*N* = 39) since numerically the SN values were also lower post procedurally; however, this difference did not reach statistical significance. Furthermore, the RFA group differed from the PFA group in several variables including age, body weight, AF recurrences, total procedural time, and other factors (Supplementary Table 2).

**Figure 7:**
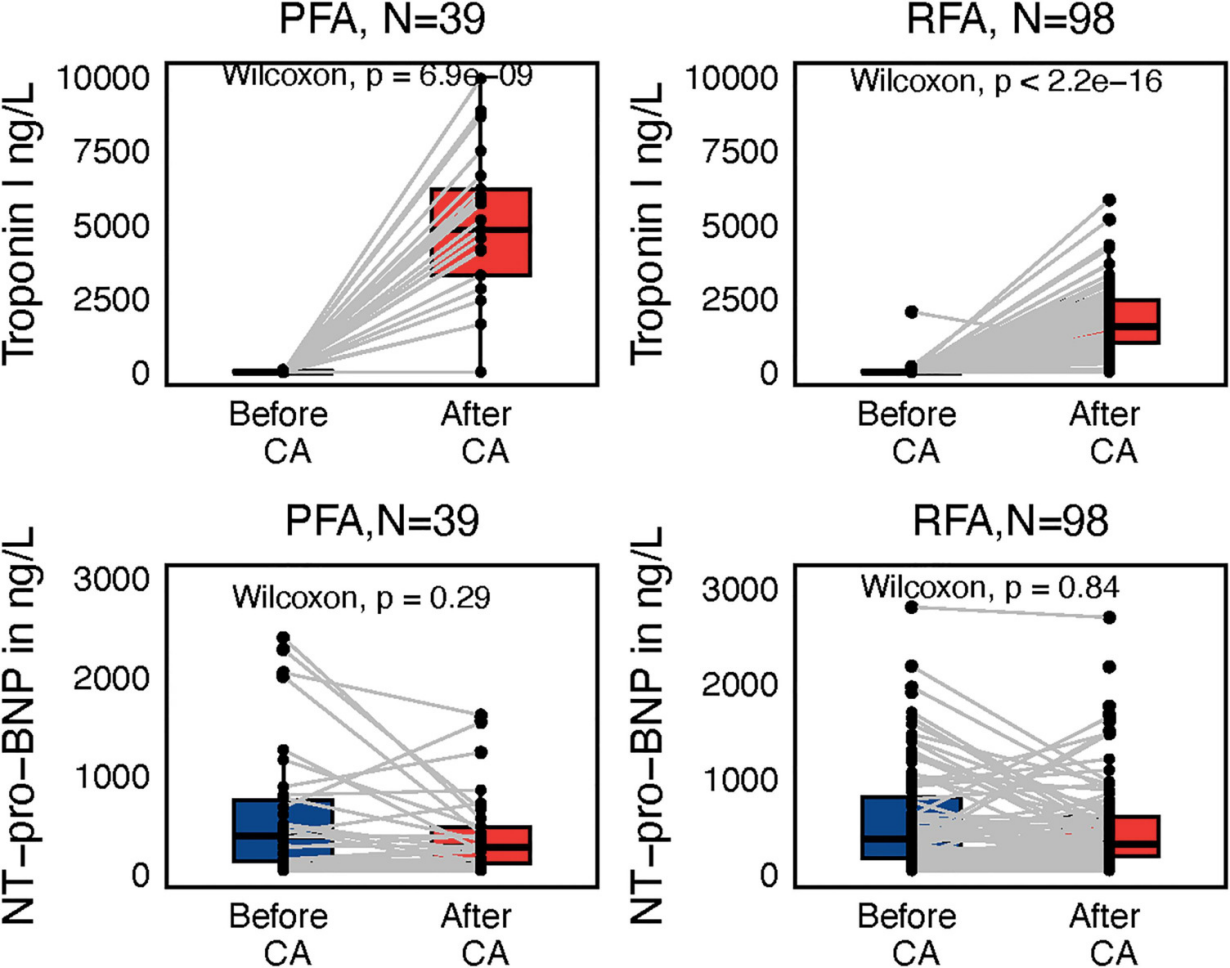
Subgroup paired Wilcoxon test of troponin I and NT-proBNP values reflecting ablation modality, either pulsed-filed ablation (PFA) or radiofrequency ablation (RFA), before and after the procedure with interconnecting lines between values before and after.

Although SN after the procedure correlated significantly with NT-proBNP plasma levels (*P* < 0.001), there was no difference between NT-proBNP before and after the procedure (Figure 7). On the contrary, Troponin I (TnI) plasma levels differed significantly before and after the procedure in both the PFA and the RFA subgroup (both *P* < 0.001); higher TnI levels after the procedure were found in the PFA group as compared with the RFA group (*P* < 0.001).

Lower TnI before the procedure was associated with AF recurrences (*P* = 0.041), but the overall Troponin levels before the procedure were very low, limiting any serious discriminative capacity in terms of AF recurrences.

Neither TnI nor NT-proBNP after the procedure have, according to our study, any relation to subsequent AF recurrences (*P* = 0.49, *P* = 0.56).

Hemoglobin and thrombocyte count dropped after the procedure as compared with their preprocedural values in the whole group (*P* < 0.001 for both). The drop in hemoglobin was comparable in the RFA and PFA groups (*P* = 0.002, *P* = 0.001, respectively). Notably, the haematocrit decreased as well (*P* < 0.001). Therefore, we assume it was more related to haemodilution associated with sheath and catheter saline perfusions than with haemolysis caused by the PFA. Variables of intravascular hemolysis were examined only in the PFA group (*N* = 39), since after hundreds of thousands of RFA procedures worldwide the chances of RF-caused hemolysis are very low.

**Figure 8:**
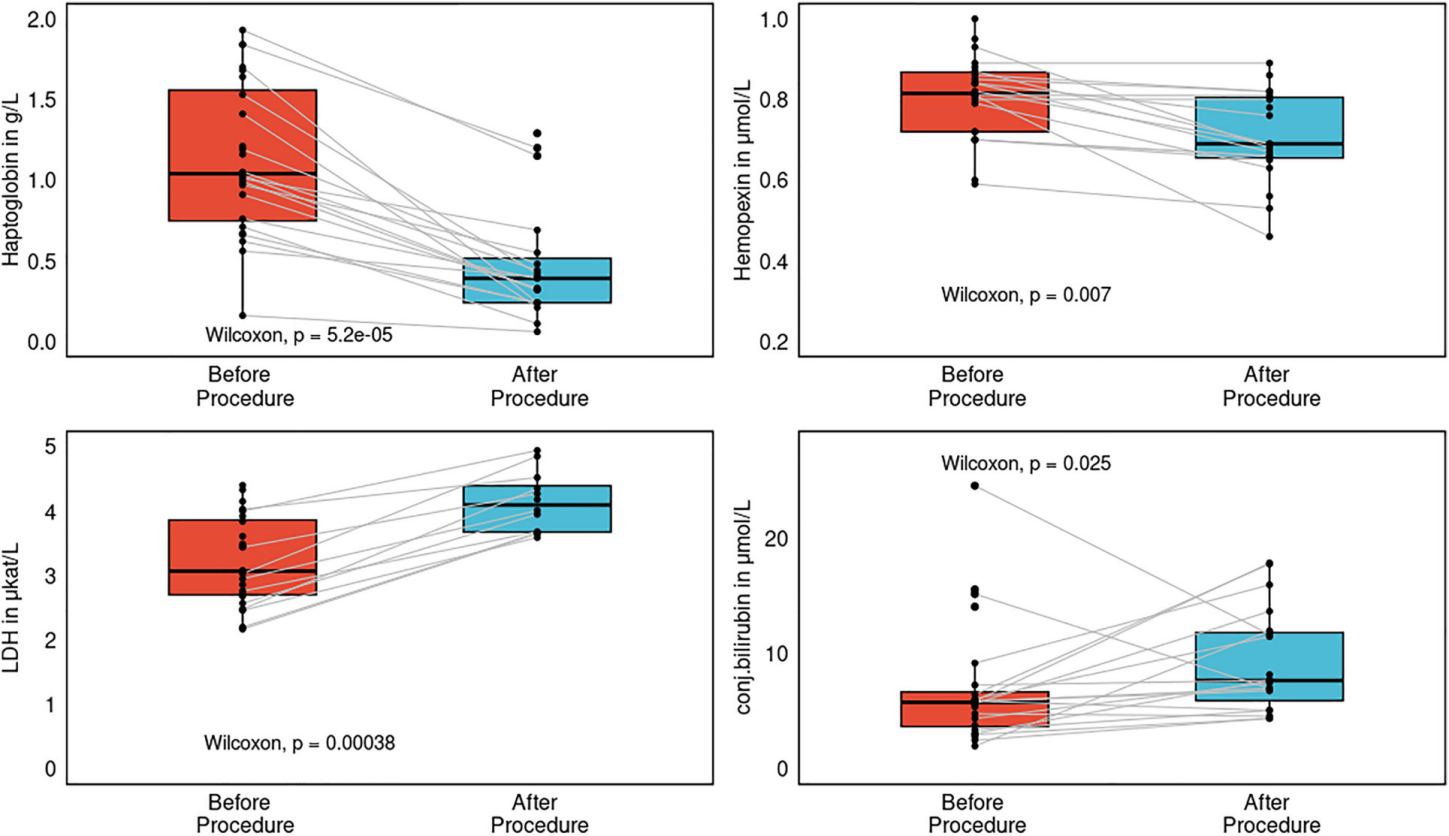
Separate subgroup analysis of the pulsed-field ablation reflecting several markers of intravascular hemolysis: lactate dehydrogenase (LDH), haptoglobin, hemopexin, and conjugated bilirubin before and after the procedure, represented as paired box plots with central jitter.

Both haptoglobin and hemopexin dropped after the procedure (*P* < 0.001*, P* = 0.007, Figure 8). LDH and conjugated bilirubin increased after the procedure (*P* < 0.001, *P* = 0.025, Figure 8). None of the patients have any clinical signs or clinical sequelae of hemolysis such as kidney injury, icterus, or subicterus. There was no association between the number of PF pulses and the magnitude of intravascular hemolysis. Also, both markers of silent brain injury, neuron-specific enolase (*N*SE) and S100B, were not increased after the procedure as compared with their plasma levels before the procedure (*P* = 0.38, *P* = 0.49, respectively). In contrast, both markers of inflammation, high-sensitivity C-reactive protein (hs-CRP) and interleukin-6 (IL-6), were increased after the procedure as compared with their levels before the procedure (*P* < 0.0001 for both). Neither IL-6 nor hs-CRP correlated with the postprocedural plasma SN levels (*P* = 0.92, *P* = 0.36).

## Discussion

The main findings of our prospective registry analysis can be summarized as follows:

1. SN levels after catheter ablation for AF were lower than before the procedure
2. Healthy individuals produce higher plasma SN levels than do patients with AF
3. SN levels do not differ between the LA and the CS
4. Lower postprocedural SN levels are very weak predictor of postablation AF episodes
5. PFA is a significantly faster method than RFA, with a lower AF recurrence rate
6. Intravascular hemolysis does occur after PFA, but without clinically relevant sequelae
7. PFA, compared with RFA, causes greater myocardial damage but a comparable inflammatory response.

### Secretoneurin and its biological effects

SN has a broad range of biological effects, including immune response, chemotaxis/inflammation, endothelium relaxation, and cell cycle regulation (5). The main intracellular effect of endogenous SN is to inhibit CaMKII; this reduces RYR2 phosphorylation and thus decreases leakage of Ca^2+^ from the sarcoplasmic reticulum (6). These effects lead to both improved excitation–contraction coupling and decreased arrhythmogenic risk in general (5, 6). In an animal model, SN treatment decreased Ca^2+^ spark frequency and dimension in cardiomyocytes during isoproterenol challenge and also reduced the incidence of Ca^2+^ waves, delayed afterdepolarizations, and spontaneous action potentials (6).

It is well known that cytosolic and mitochondrial Ca^2+^ are dysregulated in heart failure and AF (2). In contrast, mutation of the T-box transcription factor (TBX5) is associated with “lone” (without structural heart disease) AF (9). In this model situation, calcium homeostasis disruption is also one of the key explanations for arrhythmogenicity resulting from this mutation.

In an animal model, on a molecular basis, interaction between TBX5/GATA4 normalized intracardiomyocyte calcium flux and the expression of calcium channel genes. Moreover, atrial rhythm instability caused by TBX5 haploinsufficiency was balanced by a decreased dose of phospholamban, a sarco/endoplasmic reticulum Ca^2+^-ATPase inhibitor. Decreased sarcoplasmic reticulum calcium flux has a stabilizing effect on TBX5-dependent AF susceptibility (10). This is another proof that decreased calcium leakage from the sarcoplasmic reticulum has antiarrhythmic effects.

AF is sustained by a re-entrant mechanism that is partly dependent on atrial structural remodeling. Increased CaMKII activity occurs in persistent AF. CaMKII must contribute to AF development, yet electrical remodeling in AF differs considerably from the electrophysiological effects of CaMKII (11).

AF-promoting changes were studied in a dog heart failure model. In this model, CaMKII increased phosphorylation of phospholamban by 120%, enhancing SR Ca^2+^ uptake by reducing phospholamban inhibition of SR (12). Total RyR2 and calsequestrin expression were significantly reduced, by 65% and 15%, respectively, contributing to SR dysfunction (12). On the basis of all these observations, we hypothesize that SN dynamics may be associated with “calcium-dependent” arrhythmias, including AF.

### Secretoneurin in clinical situations

SN has been studied in several clinical scenarios, most of them very acute, life-threatening situations. However, only two of them were associated with arrhythmias. In an animal model, myocardial pro-SN expression was increased in CPVT mice, and SN overexpression by a viral vector attenuated arrhythmia induction (6). SN was also increased in CPVT patients irrespective of ventricular tachycardia episodes and after out-of-hospital cardiac arrest, after which plasma SN levels declined (6). In patients who underwent coronary artery bypass graft (CABG) surgery, SN levels were significantly higher in nonsurvivors in comparison with survivors, both before (168 vs. 160 pmol/L) and after (173 vs. 143 pmol/L) surgery (13). Adding postoperative SN concentrations to the current evaluative standard (the European System for Cardiac Operative Risk Evaluation II) also improved its risk stratification (13). In patients with aortic stenosis, a cutoff of 204 pmol/L predicted mortality in this cohort also (14). Lower creatinine clearance and use of diuretics has been associated with increased SN concentrations (14). In contrast, no association with cardiac biomarkers (NT-proBNP, troponin T) was observed in either of these studies (13, 14). Moreover, in critically ill patients with respiratory infections in the intensive care unit, SN was able to predict mortality on top of classical risk factors, and no association with NT-proBNP was observed (15). The optimal cutoff to predict mortality in this ICU setting was 175 pmol/L (15). However, studies published up to 2019 have all used an in-house radioimmunoassay (RIA) to measure SN. ELISA and RIA methods are not directly comparable: RIA yields much higher plasma SN levels than does ELISA. In more than 800 healthy individuals, SN concentrations were 58.6 (IQR 57.1–62.1) pmol/L after exclusion of structural heart disease by echocardiography. Moreover, women and the elderly in general had somewhat higher SN levels than those of the rest of the cohort (16). These values are also comparable to those in our own sample of healthy individuals (8). In a subanalysis in the GISSI-HF trial, SN concentrations upon randomization stratified patients with poor and favorable prognoses (17). SN concentrations (mean age 67 y, 80% male) were 42.6 (35–62.8 pmol/L) on randomization and 42 (34.5–53.1) pmol/L after 3 months, which are lower than SN concentrations in healthy subjects (in the original publication they were higher, but this no longer holds true) (16, 17). SN concentrations were higher in patients with ACS compared with patients with chest pain without ACS: median 32.8 (IQR 27.5–42.8) vs. 28 (24.5–34) pmol/L (18). Patients with ECG changes reflective of acute myocardial infarction had higher SN concentrations. Both values are lower than SN concentrations in healthy individuals (8, 16). In our previous study, healthy individuals produced higher SN plasma levels than those in patients with ischemic or dilated cardiomyopathy (8). These observations are in line with our recent results: patients with AF, whether before or after catheter ablation, also have lower plasma SN levels than healthy individuals. Now it is confirmed in these registry data, in our previous study, in the GISSI-HF subanalysis, and in patients with chest pain that lower plasma SN levels are associated with different diseases as compared with higher values in healthy individuals. We may speculate that during the acute phases of a particular disease, the plasma SN level increases to compensate mainly for calcium dysregulation due to RYR2 hyperphosphorylation. After the risk of arrhythmia or the arrhythmia itself wanes, the plasma SN level declines. Since our registry data are the very first regarding SN dynamics in the AF patient population, we have no other AF-specific population with which to compare it. We may hypothesize that after radiofrequency catheter ablation, SN is decreased due to a specific tissue response to this kind of ablation. In contrast, the PFA group was considerably smaller and this may have prevented the difference from reaching significance.

### Inflammatory markers and cell death

The character of cell death differs significantly among different methods of ablation (19). RFA uses radiofrequency currents to generate high temperature to cause thermal damage to the tissue, while PFA uses strong electric fields to disrupt the phospholipid bilayer integrity of cell membranes (19). Cardiomyocytes have the lowest electroporation threshold of all tissues, which decreases collateral damage, especially to the phrenic nerve and esophagus. RFA denatures proteins, and this is quickly followed by cytotoxicity that causes coagulative necrosis. PFA, in contrast, being a nonthermal energy source, most commonly kills cells by inducing apoptosis (20). Moreover, the injury zone after PFA is well delineated and clearly bounded by the surrounding tissue (21).

Inflammation markers such as hs-CRP are increased after RFA, comparably to cryoablation (22). A detailed histological analysis after 7 and 30 days showed that PFA produced a shorter tissue inflammatory response than RFA (23). Another study showed tissue necrosis, inflammatory response, and fibrosis almost exclusively in all tissue sections after RFA as compared with PFA, and the authors speculated that RFA causes greater myocardial damage and inflammation than PFA (24). Our registry data have shown that myocardial injury is more pronounced in PFA than in RFA, but the inflammatory response is comparable between these two methods.

### Hemolysis related to PFA

Clinical trials have demonstrated that PFA is a promising and highly effective method with an excellent safety profile (25, 26). Worldwide, more than 17,000 procedures have been performed with very rare complications, as documented in the MANIFEST-17 K prospective registry (27). Renal failure requiring dialysis was reported in only 5 patients (0.03%), as presented recently at the American Heart Association meeting (28). Recently Vernier et al. reported acute kidney injury after PFA for AF in 2 cases out of 68; these patients, however received in excess of 124 and 176 applications, and the number of applications was correlated with markers of hemolysis (29). Similarly, in the MANIFEST-17 K survey, a patient in acute need of dialysis received 143 + 27 PF energy applications. In our registry, the number of PF applications was 59 ± 39, and neither clinical signs of intravascular haemolysis nor clinically relevant kidney injury were observed. Intravascular hemolysis was present as detected by the hemolysis markers LDH, haptoglobin, hemopexin, but completely without clinical sequelae. It seems prudent to limit the number of PF applications, although at this time we do not have a specific number of safe applications. Furthermore several factors may increase the risk of intravascular hemolysis in general, namely poor tissue contact, pre-existing renal failure, and kidney hypoperfusion due to either on-going arrhythmia, heart failure, or both. LDH is an important enzyme of the anaerobic metabolic pathway and a marker of cell lysis—red blood cells are rich in LDH, thus plasma LDH levels are increased in intravascular haemolysis (30). Plasma LDH levels will also increase due to myocardial cell death as a result of catheter ablation. Haptoglobin is an acute phase protein and its major role is to bind free hemoglobin to prevent iron loss and renal damage (31). Therefore, with hemolysis the plasma pool will decrease, as documented in our own results. Hemopexin is able to bind free heme and carry it to the liver, where it is internalized and degraded, thus preventing heme-mediated oxidative stress and iron loss (32). Haptoglobin and hemopexin levels were significantly decreased after the PFA procedure in comparison with their preprocedural values in our cohort. Hemolysis was studied only in the PFA group and there is no link between SN alterations and hemolysis. Also the sample size is too low to perform a valid subgroup analysis.

For none of these markers is there an exact definition or range indicating intravascular haemolysis. However, elevation of LDH with concomitant declines in both haptoglobin and hemopexin in the absence of acute inflammation may be specific to intravascular hemolysis.

Recommended Follow-up studies with multicenter validation, standardization of detection protocols, and interventional study designs are necessary to confirm our unicentric observational study results. Current evidence is insufficient to support SN as a clinical decision-making biomarker.

### Limitations

This is a unicentric study with a limited sample size, especially in the healthy control group. In the AF group male prevalence and more frequent RFA than PFA were present. There is also significant age disparity between the healthy individual group and the AF group. Blinded assessment procedures were not undertaken. The follow-up is as yet quite short and the total AF recurrences are therefore not numerous.

## Conclusion

Plasma secretoneurin levels decrease after catheter ablation for AF, and patients with AF produce lower SN levels than do healthy individuals. Lower postprocedural levels of SN are moderately associated with AF recurrences. Healthy individuals produce higher plasma SN levels than patients with AF. PFA is a significantly faster method than RFA, with a lower AF recurrence rate. Intravascular hemolysis does occur after PFA, but without clinically relevant sequelae. However, in comparison with RFA, PFA causes greater myocardial damage but a similar inflammatory response.

## Data Availability

The data analyzed in this study is subject to the following licenses/restrictions: the data that support the findings of this study are available from the corresponding author upon reasonable request and in compliance with the General Data Protection Regulation. Requests to access these datasets should be directed to jiri.plasek@fno.cz.
