# Supplementary figures and images for: Secretoneurin plasma levels are decreased after catheter ablation for atrial fibrillation—patients with AF produce lower SN levels than healthy individuals: the SAFE registry
Source: Front Cardiovasc Med. 2025 Oct 16;12:1664855. doi: 10.3389/fcvm.2025.1664855 (PMC12571760; doi:10.3389/fcvm.2025.1664855)

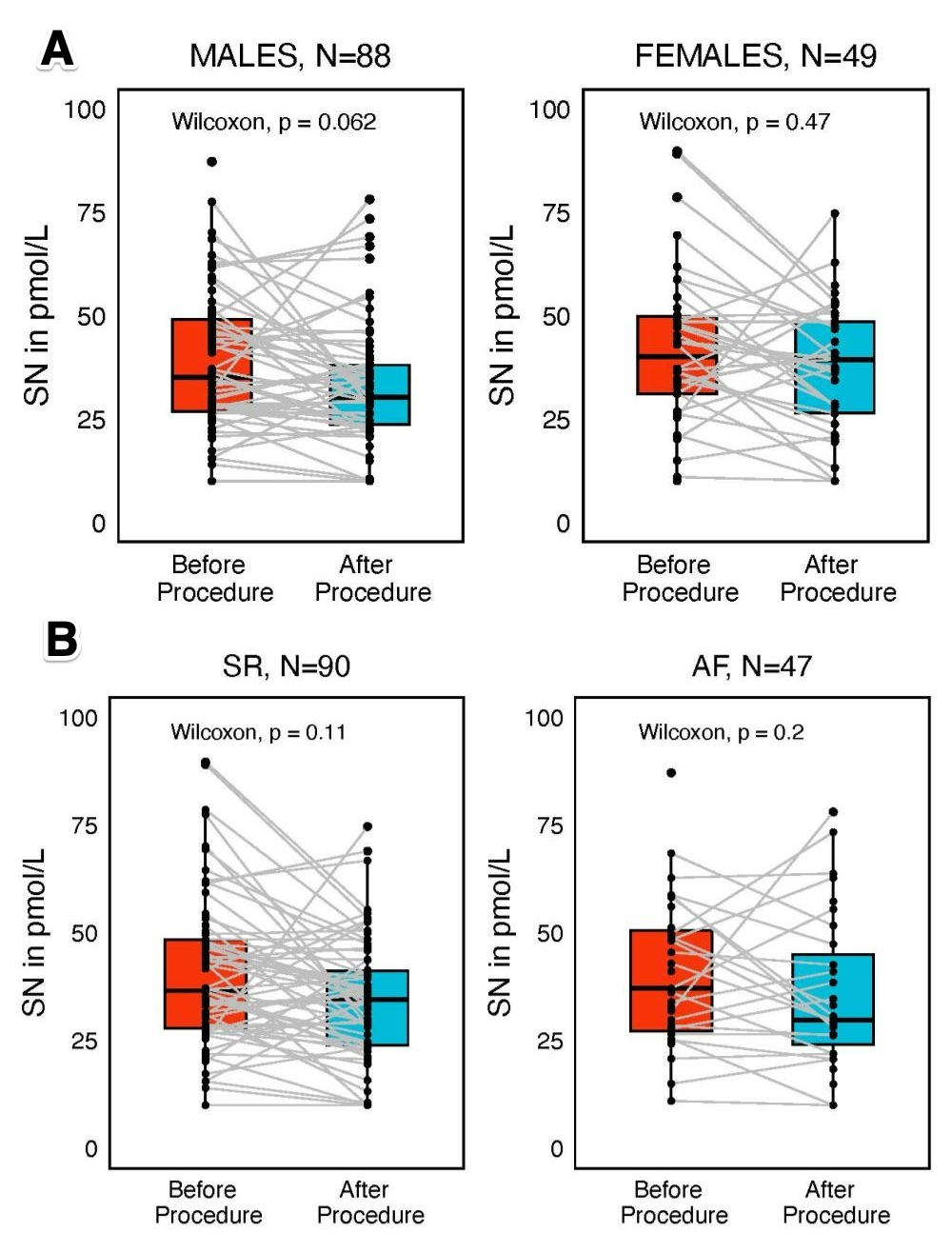

Supplement: Supplementary file 1 [file Datasheet1.zip › Supplementum TIFF/S2AB.tiff]

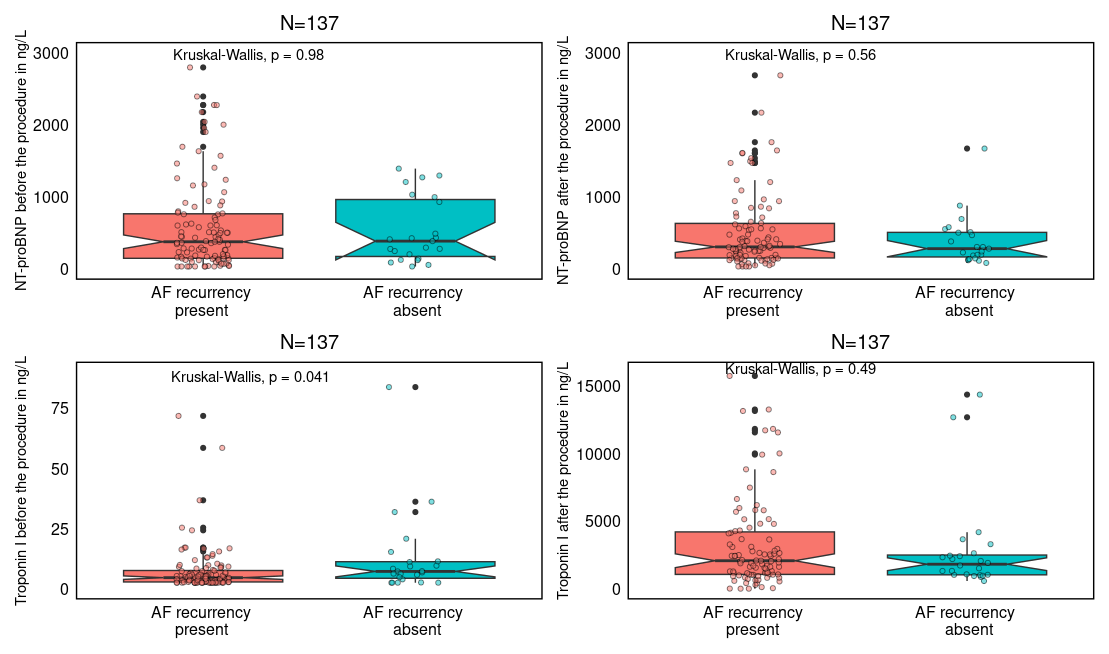

Supplement: Supplementary file 1 [file Datasheet1.zip › Supplementum TIFF/S6.tiff]

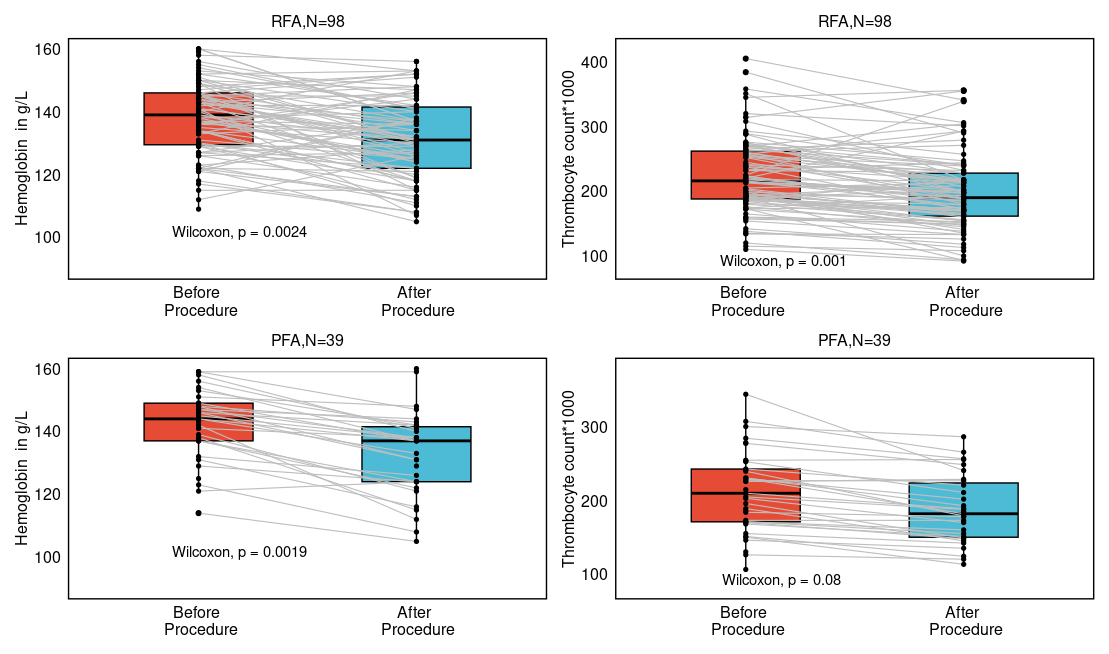

Supplement: Supplementary file 1 [file Datasheet1.zip › Supplementum TIFF/S7.tiff]

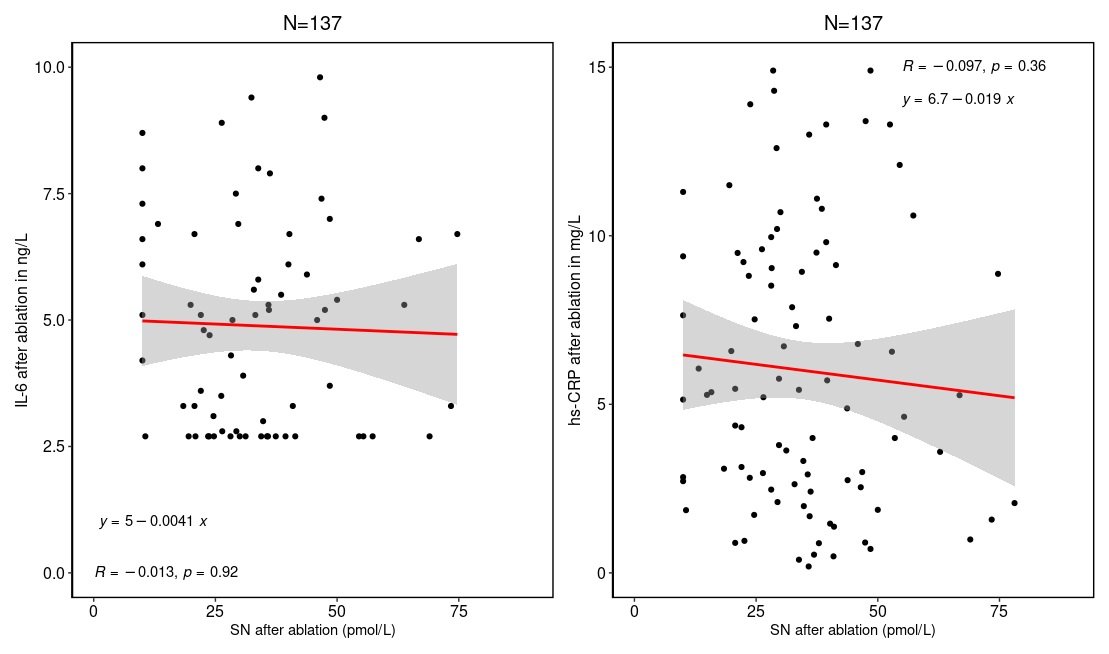

Supplement: Supplementary file 1 [file Datasheet1.zip › Supplementum TIFF/S11.tiff]

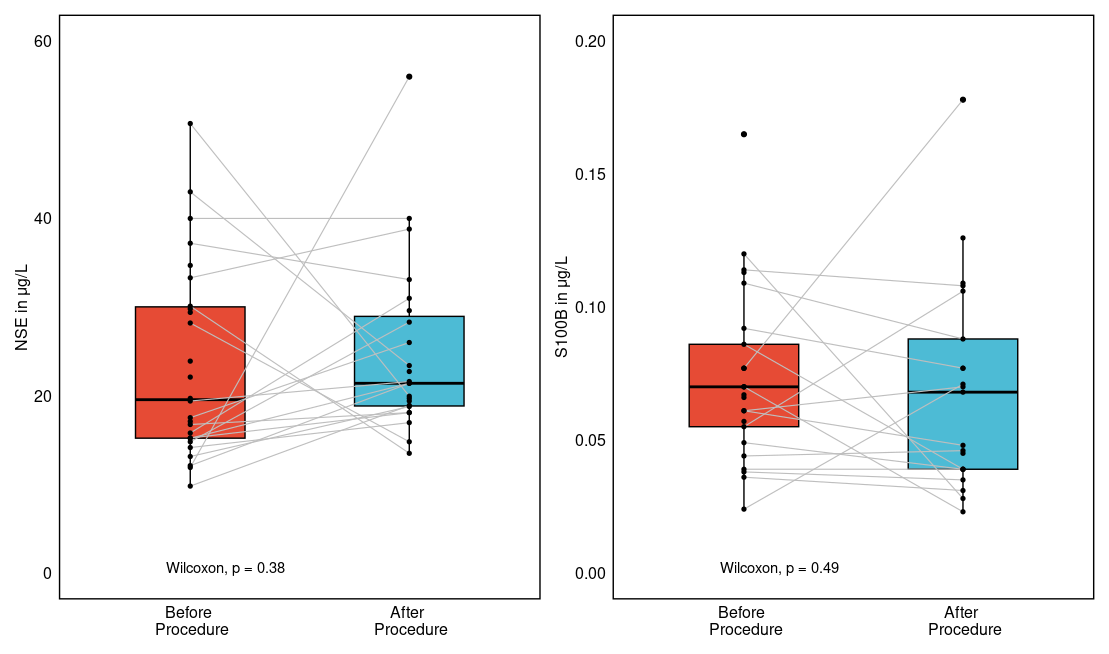

Supplement: Supplementary file 1 [file Datasheet1.zip › Supplementum TIFF/S8.tiff]

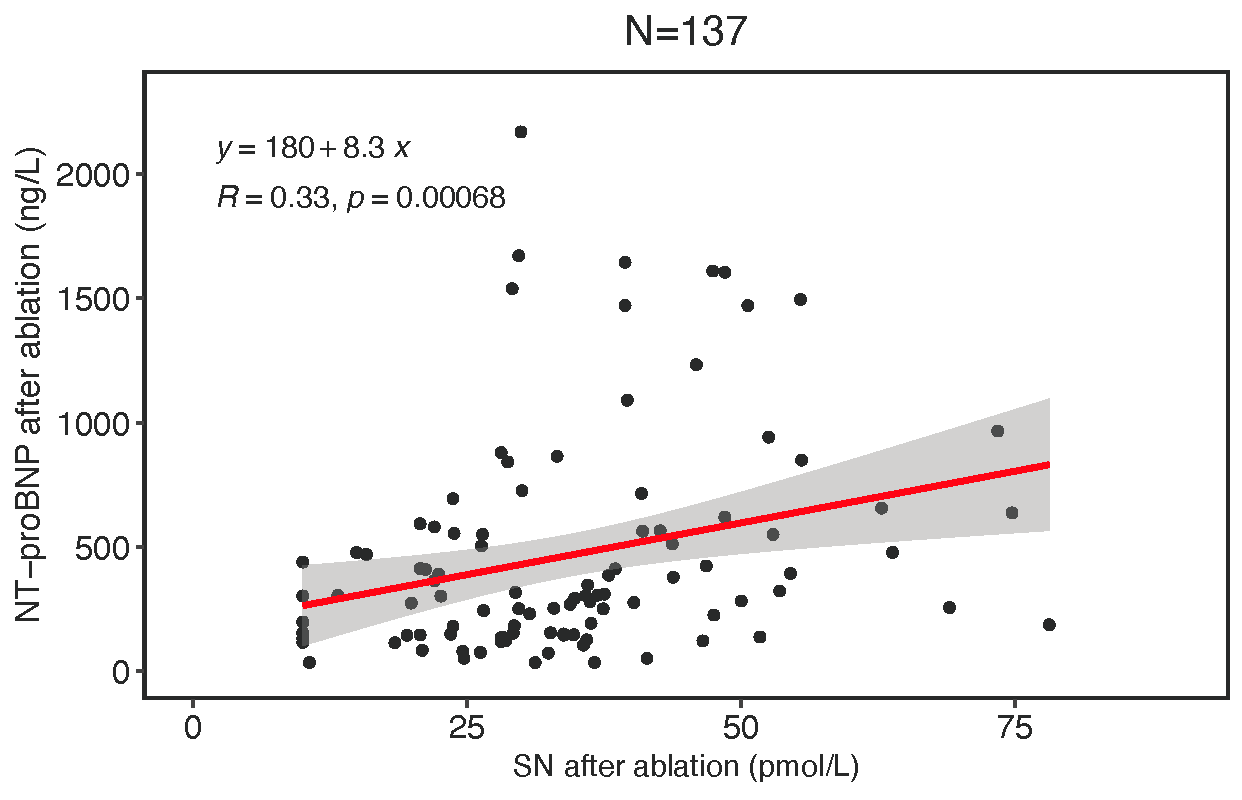

Supplement: Supplementary file 1 [file Datasheet1.zip › Supplementum TIFF/S4.tiff]

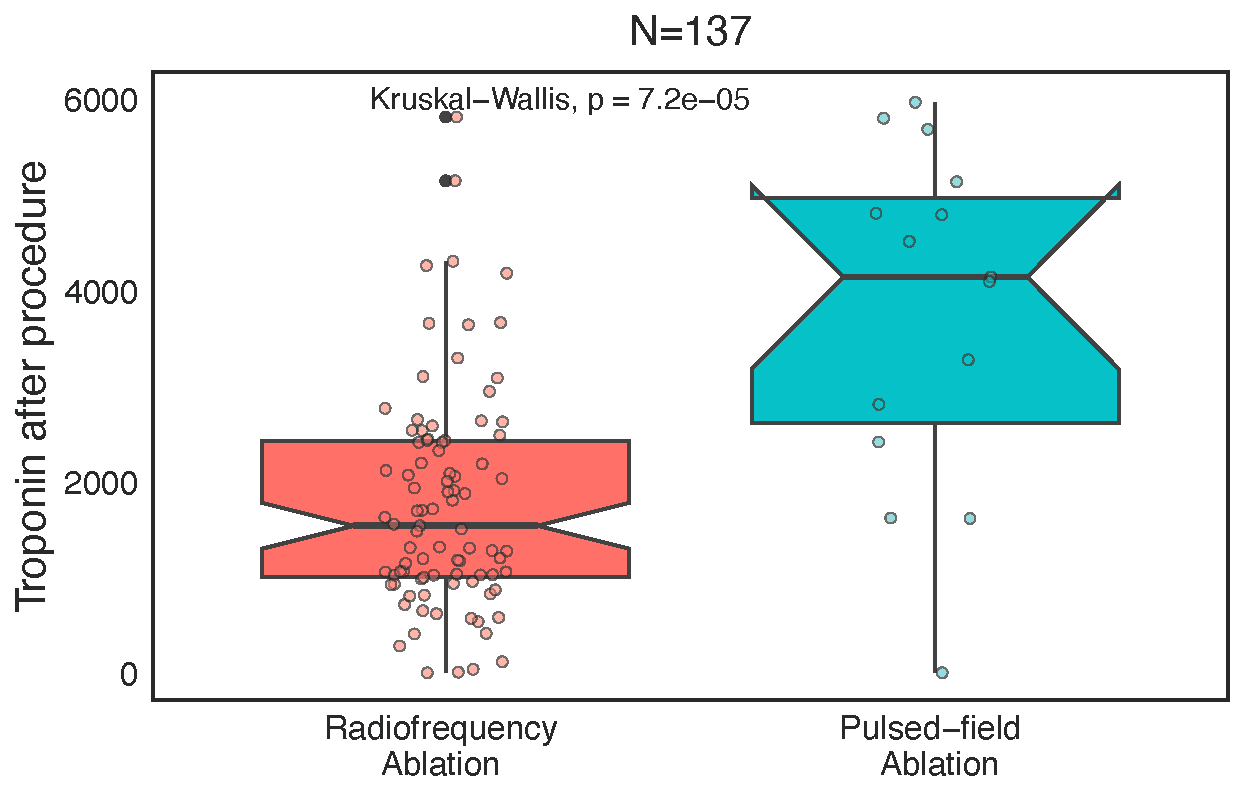

Supplement: Supplementary file 1 [file Datasheet1.zip › Supplementum TIFF/S5.tiff]

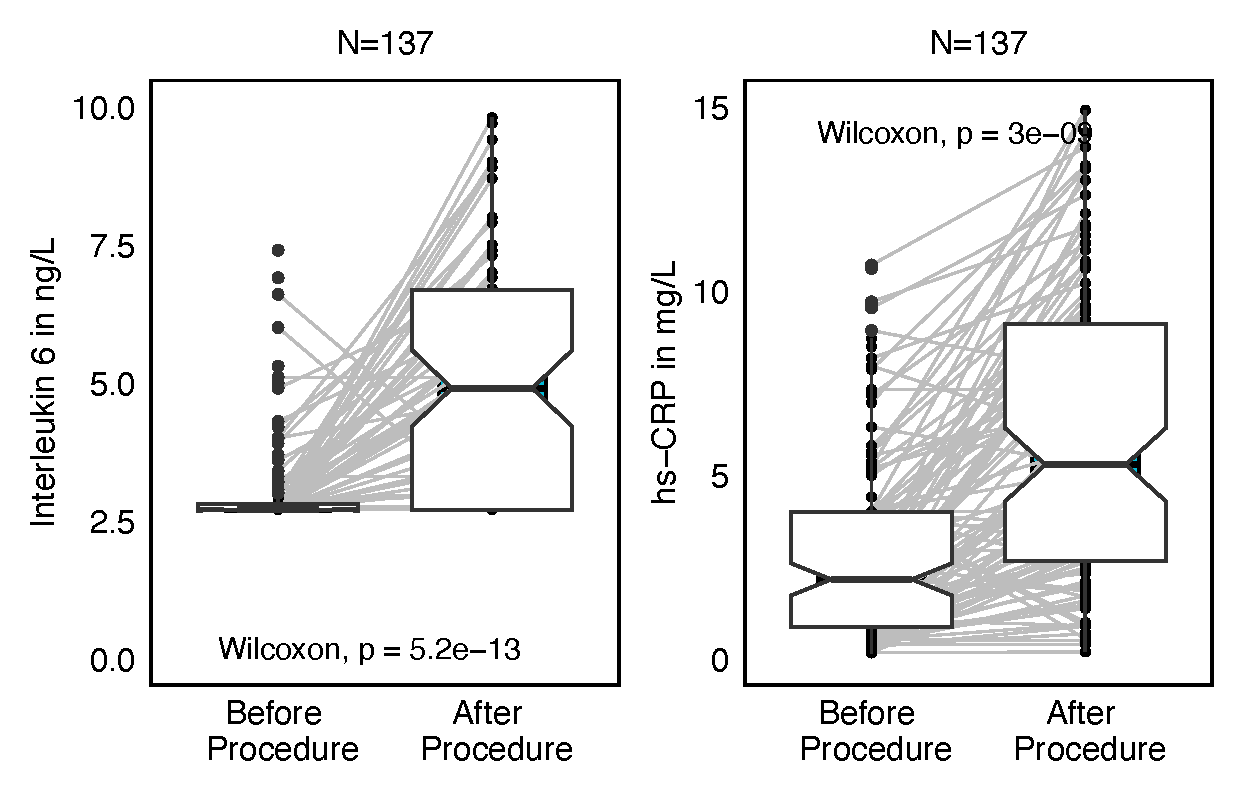

Supplement: Supplementary file 1 [file Datasheet1.zip › Supplementum TIFF/S9.tiff]

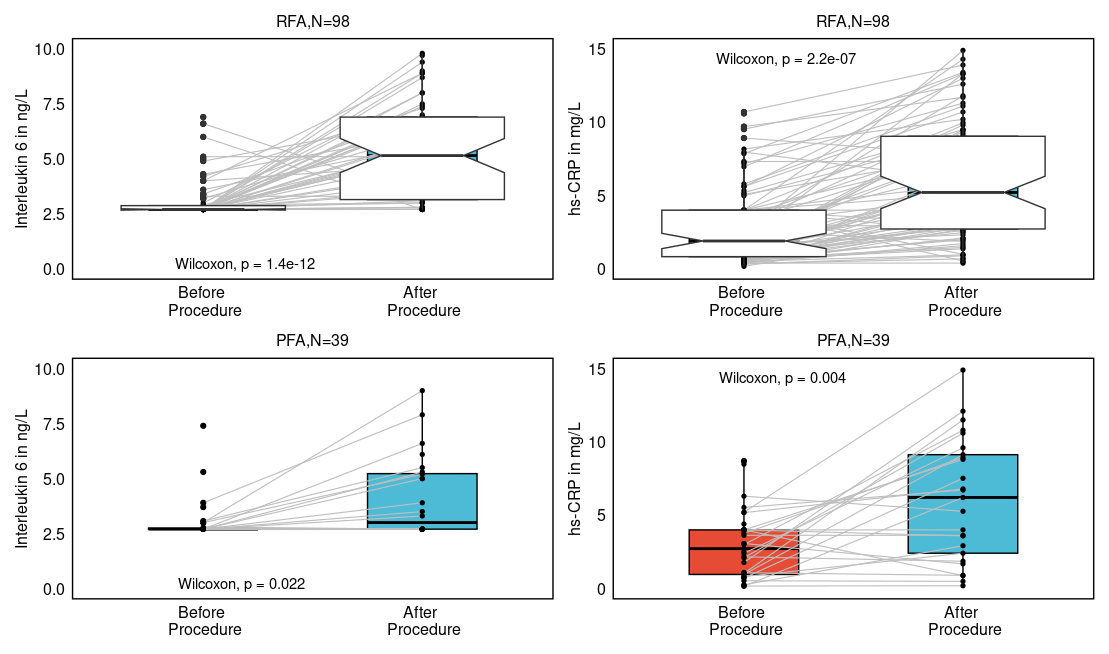

Supplement: Supplementary file 1 [file Datasheet1.zip › Supplementum TIFF/S10.tiff]

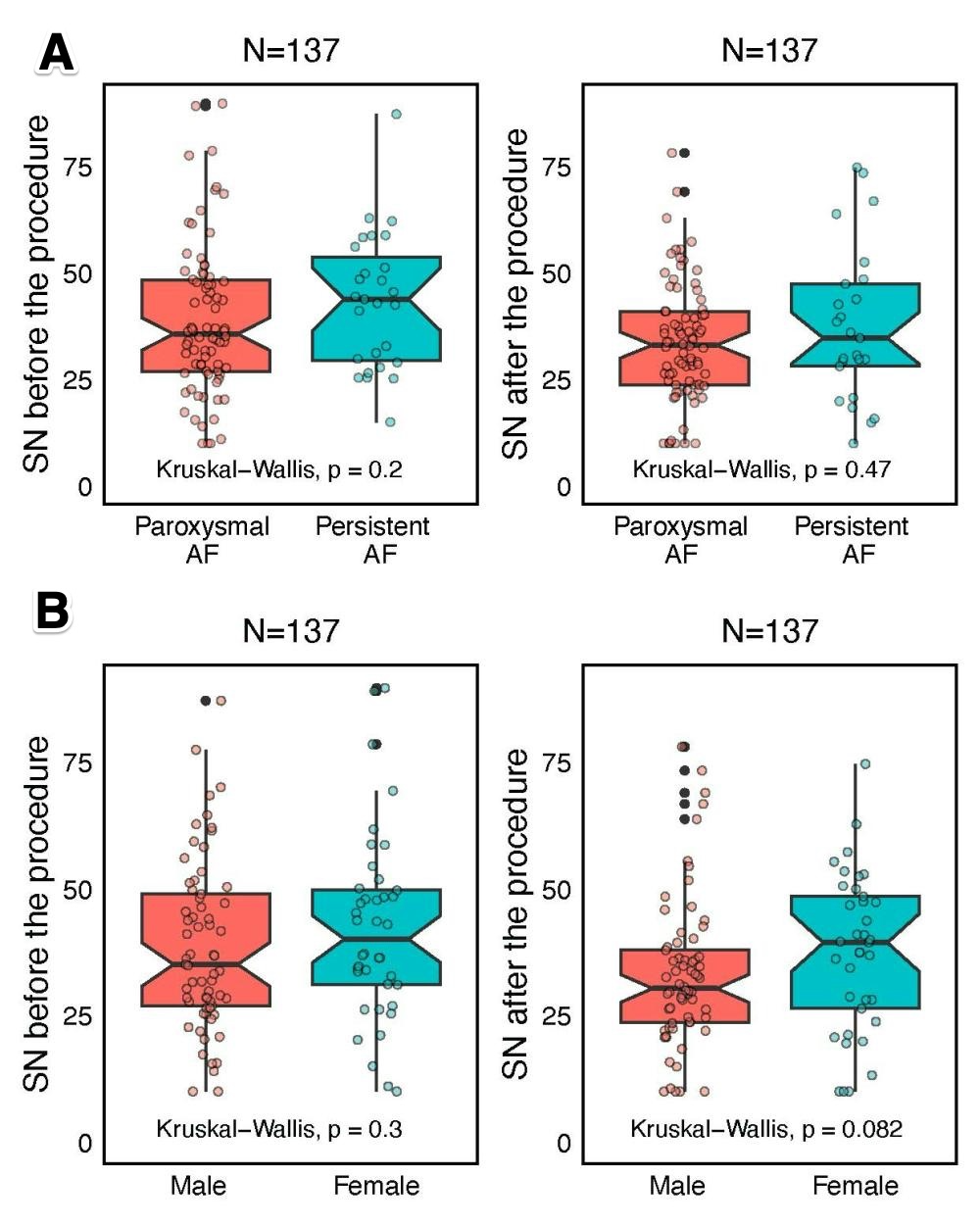

Supplement: Supplementary file 1 [file Datasheet1.zip › Supplementum TIFF/S1AB.tiff]

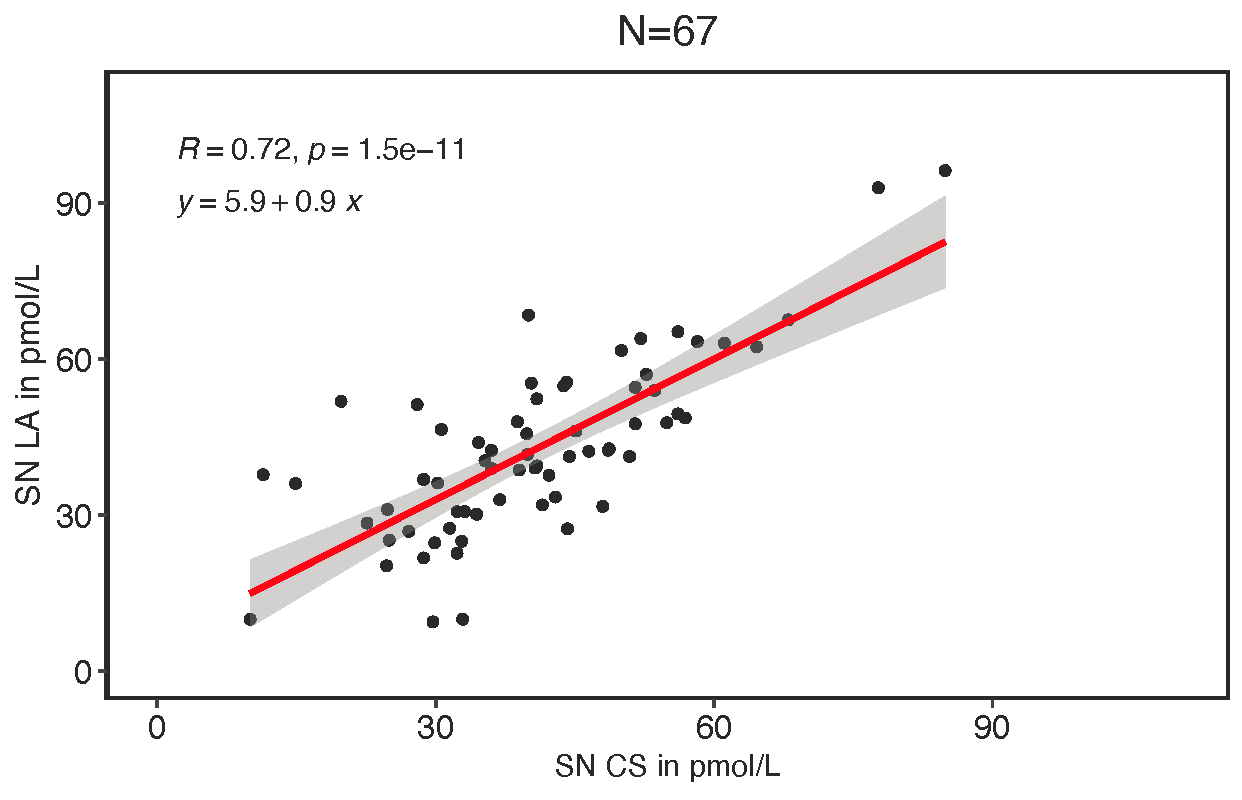

Supplement: Supplementary file 1 [file Datasheet1.zip › Supplementum TIFF/S3.tiff]
